# Homologous recombination deficiency in breast cancer: genomic characteristics, clinical implications, and predictive value in neoadjuvant therapy

**DOI:** 10.3389/fonc.2025.1608968

**Published:** 2025-10-06

**Authors:** Jing Huang, Feiyang Luan, Li Yan, Xiao Liang, Xinyue Ma, Yiyang An, Sirui Hu, Guoqiang Gao, Yuanyuan Wang, Jin Yang, Danfeng Dong

**Affiliations:** ^1^ Department of Oncology, The First Affiliated Hospital of Xi’an Jiaotong University, Xi’an, China; ^2^ Department of Urology, The First Affiliated Hospital of Xi’an Jiaotong University, Xi’an, China; ^3^ Department of Pathology, The First Affiliated Hospital of Xi’an Jiaotong University, Xi’an, China

**Keywords:** homologous recombination deficiency, triple negative breast cancer, genomic scar score, neoadjuvant therapy, breast cancer, clinical characteristics

## Abstract

**Background:**

Homologous recombination deficiency (HRD) significantly influences breast cancer development. HRD-positive breast cancer is more sensitive to DNA-targeting cytotoxic drugs, and may benefit from incorporating platinum-based agents in neoadjuvant therapy. However, standardized HRD phenotyping in China remains unclear, and research on the clinical pathological features of HRD-positive breast cancer is limited. Furthermore, its predictive value for neoadjuvant therapy efficacy is uncertain.

**Methods:**

We employed the AmoyDx HRD kit to assess HRD status in a cohort of 133 Chinese breast cancer patients from the First Affiliated Hospital of Xi’an Jiaotong University. Differences in genomic features, clinical characteristics, and neoadjuvant therapy outcomes between HRD-positive and HRD-negative patients were evaluated.

**Results:**

There were 54.1% of patients exists HRD-positivite status. TP53 mutations were the most frequent among homologous recombination repair (HRR) pathway genes, showing significant differences between HRD-negative and HRD-positive groups (*P* = 0.004). HRD-positive had higher T stage, lower ER/PR/AR expression, higher Ki67 index, and a higher incidence of triple-negative breast cancer (TNBC) (all *P* < 0.05). TNBC had a higher GSS score than Luminal A patients (*P* = 0.001). Tumors with higher GSS scores were more likely to have low ER (*P* = 0.001), PR (*P* = 0.002) and high Ki67 expression (*P* = 0.001). There was no statistically significant difference in the efficacy of neoadjuvant therapies between HRD-positive and HRD-negative groups (*P* = 0.158). However, HRD-positive TNBC patients had a higher pathologic complete response (pCR) rate with anthracycline-based regimens (*P* = 0.042). No significant difference was observed in the proportion of patients experiencing progression between HRD groups (*P* = 0.458).

**Conclusion:**

Using a GSS-based HRD detection method, we characterized HRD genomic features, highlighting TP53 mutations and clinical-pathological associations. HRD-positive patients, especially those with high GSS scores, had lower ER/PR and higher Ki67 expression. TNBC had a higher HRD-positive rate.The role of HRD detection in predicting the efficacy of neoadjuvant therapy for breast cancer patients needs further clinical verification. In patients with TNBC, the HRD status had no significant impact on the efficacy of platinum-containing neoadjuvant therapy. However, adding anthracyclines improved outcomes for HRD-positive TNBC. This research helps establish Chinese-specific HRD detection standards and support individualized treatment strategies.

## 1 Introduction

Breast cancer is the most prevalent malignancy among women globally. According to the American Cancer Society (ACS), breast cancer accounts for 32% of all newly diagnosed cancer cases in American women, with a mortality rate of 15%, second only to lung cancer (1). Notably, the incidence rate among women under 50 increased by 1.1% from 2012 to 2019, significantly surpassing the 0.5% rise in women aged 50 and above, suggesting earlier disease onset. China faces similar challenges, with rising incidence and mortality rates making breast cancer the most common malignancy among Chinese women (2). Triple-negative breast cancer (TNBC), comprising 15%-20% of cases, is characterized by aggressive proliferation, high malignancy, and a propensity for recurrence and metastasis. These patterns underscore the urgent need for precision diagnostics and therapeutics (3).

Research indicates that approximately 10% of breast cancer cases exhibit distinct genetic predispositions or unique genomic features (4). Genomic instability, a hallmark of cancer, involves the homologous recombination repair (HRR) pathway, which plays a pivotal role in DNA damage response (DDR) (5). Mutations, deletions, or methylation of HRR-related genes can lead to homologous recombination deficiency (HRD), a phenotype that impairs the repair of DNA double-strand breaks (6). HRD is implicated in several cancers, including endometrial (34.4%), ovarian (20.0%), breast (15.6%), and pancreatic cancers (15.4%) (7). BRCA1 and BRCA2 are critical mediators in the HRR pathway. The germline BRCA mutation (gBRCAm) rate in TNBC is 11.2%, markedly higher than the 5.3% observed in the broader breast cancer population (8), presenting opportunities for targeted therapy. These findings emphasize the clinical importance of BRCA testing for high-risk patients with elevated metastatic potential (9).

Tumor cells with HRD phenotypes are more sensitive to DNA-damaging agents, positioning HRD as a promising therapeutic target. Platinum-based drugs, through purine base alkylation and intra-/inter-strand crosslink formation, show enhanced efficacy against HRD-positive tumors (10). PARP inhibitors (PARPi) exploit synthetic lethality: while BRCA-deficient cells rely on PARP-mediated single-strand break repair, PARPi treatment blocks both repair pathways, resulting in tumor cell death (11). Initially approved for ovarian cancer, PARPis have demonstrated significant progression-free survival (PFS) benefits in BRCA1/2-mutated breast cancer patients in trials like OlympiAD and OlympiA, leading to FDA approval (12). Beyond BRCA1/2 mutations, defects in other HRR genes (ATM, PALB2, RAD51) can create “BRCAness” phenotypes (13), necessitating comprehensive HRD assessment. Emerging evidence from SWOG S1416 shows improved PFS (5.7 vs. 4.3 months) with cisplatin/veliparib in BRCA-negative but HRD-positive patients (14), while GeparSixto demonstrates higher pathological complete response (pCR) rates in HRD-positive tumors regardless of carboplatin use (15). These findings support HRD testing beyond BRCA analysis.

Anthracycline-based chemotherapy has always been the standard regimen for the neoadjuvant treatment of TNBC (16). Anthracyclines destabilize DNA, resulting in the obstruction of DNA repair, which in turn inhibits the proliferation of tumor cells. This may be the biological mechanism through which patients with TNBC benefit (17). For breast cancer patients with wild-type BRCA1/2 genes, the most commonly used treatment regimen in the neoadjuvant treatment stage is a chemotherapy regimen based on anthracyclines or taxanes. Although the anthracycline-based treatment regimen does have a relatively high response rate, it is also accompanied by a higher recurrence rate and a lower overall survival rate. Moreover, this kind of drug may also trigger acute toxic reactions, such as irreversible cardiotoxicity, myelotoxicity, alopecia, nausea, and vomiting, which limits its application (18).

The Genomic Scar Score (GSS) provides a novel approach for assessing HRD (19). HRD induces characteristic genomic alterations, including loss of heterozygosity (LOH), telomeric allelic imbalance (TAI), and large-scale state transitions (LST), which collectively quantify HRD status (20). The Chinese Expert Consensus on HRD Testing recommends SNP-based genomic scar analysis to identify patients likely to benefit from PARP inhibitors (PARPi). Although FDA-approved tests like Myriad myChoice CDx (GIS ≥42) and FoundationFocus™ CDx BRCA LOH (LOH ≥16%) are available, their application in China is limited due to unique molecular features of Chinese breast cancers. For example, TP53 mutation rates in TNBC reach 49.9%, significantly higher than the 25% seen in Western populations, with limited predictive value of non-BRCA HRR gene variants (e.g. ATM, PALB2 et al.) (21). In 2024, China’s National Medical Products Administration (NMPA) included AmoyDx’s HRD detection kit in its special review for innovative medical devices, paving the way for the first regulatory-approved HRD test in China (22). This kit, which includes TP53 and China-specific SNP markers, shows 88.6% concordance with Myriad and reduces the testing turnaround time from 17–25 days to 5–9 days (23), offering significant advances in precision oncology.

We employed the AmoyDx HRD kit to assess HRD status in a cohort of Chinese breast cancer patients from a single center in China. By analyzing the genomic characteristics of HRD in Chinese breast cancer patients, we found that TP53 mutations play a critical role in determining HRD status. Additionally, we described and compared the clinicopathological features, neoadjuvant treatment responses, family history, and disease progression between HRD-positive and HRD-negative patients. Interestingly, our findings suggest that HRD testing may serve as a valuable predictor of response to anthracycline-based neoadjuvant therapy in patients with TNBC. By integrating BRCA1/2 mutation analysis with GSS, the study aims to provide key clinical evidence for establishing HRD testing standards tailored to Chinese populations and to inform personalized treatment strategies for breast cancer.

## 2 Materials and methods

### 2.1 Patients and samples

**Figure 1:**
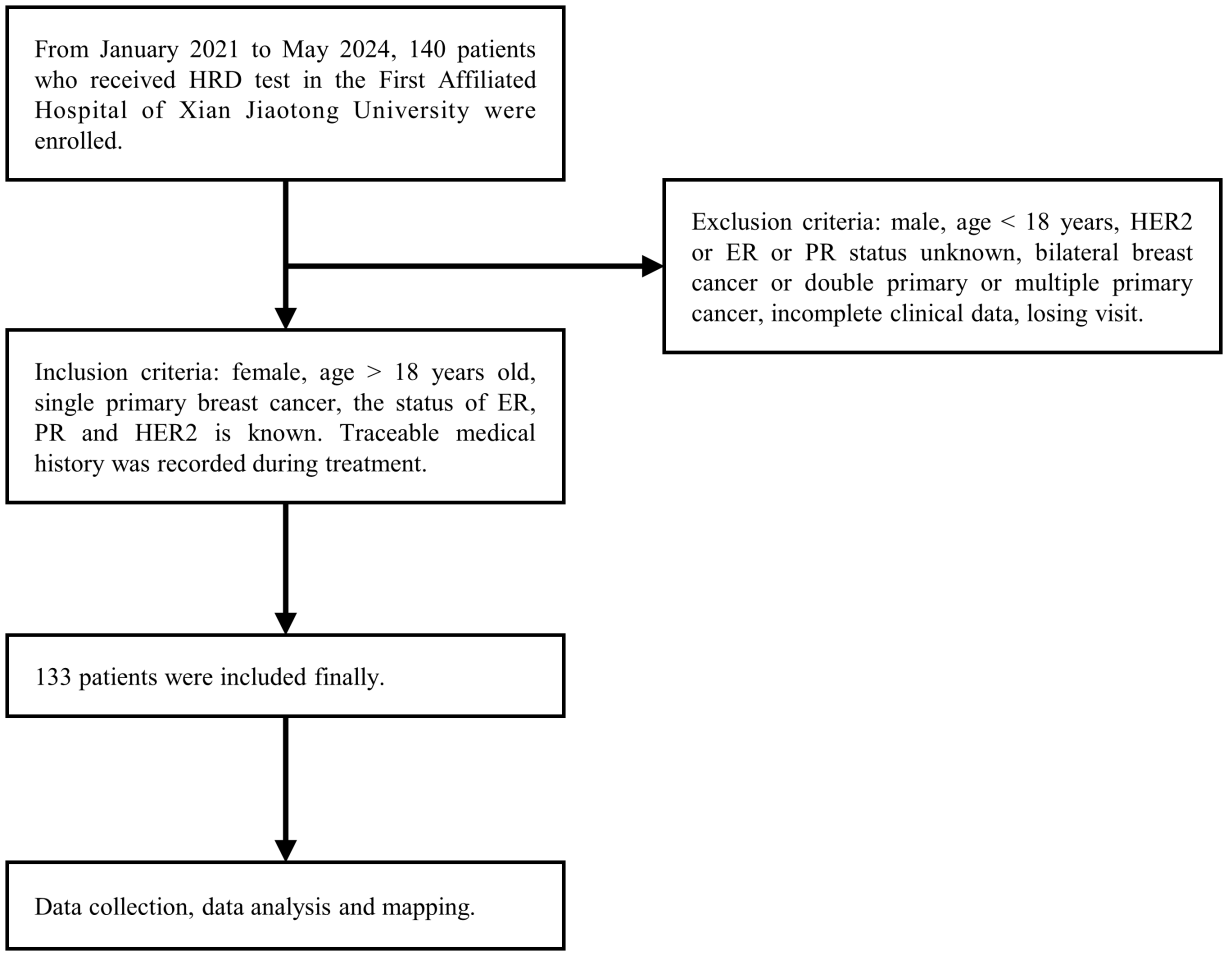
Flow chart of this study.

This study was approved by the Ethics Committee of the First Affiliated Hospital of Xi’an Jiaotong University (The approval number: KYLLSL-2021-547). Excluded were cases with incomplete data (n = 3), cases with lost to follow-up (n = 4), and cases of double primary tumors (n = 0). A total of 133 patients diagnosed with breast cancer at the First Affiliated Hospital of Xi’an Jiaotong University between January 1, 2021 and May 31, 2024 were included. All patients provided written informed consent. Clinical and pathological data were extracted from medical records and pathology reports, ensuring accuracy and validity (**Figure 1**). Immunohistochemistry (IHC) evaluations were independently conducted by two qualified pathologists in a blinded manner, and any discrepancies were resolved through joint re-evaluation. According to the 13th St. Gallen Consensus, ER and PR positivity thresholds were set at 1%. HER2 status was determined by IHC: scores of 0/1+ were HER2-negative, 3+ were HER2-positive, and 2+ required FISH testing. Samples with HER2 amplification were classified as positive, while those without were negative (24).

### 2.2 HRD detection based on genomic scar scoring

HRD detection was performed using the Illumina NextSeq CN500 high-throughput sequencing (NGS) platform, with the reference genome GRCh37/hg19 (25). Library preparation was carried out using the AmoyDx HRD Detection Kit (high-throughput sequencing method). All sample processing, library construction, sequencing, and analysis were performed in the Molecular Room of the Pathology Department at the First Affiliated Hospital of Xi’an Jiaotong University. The scope of homologous recombination repair (HRR)-related gene detection encompassed mutations in the coding regions and intron-exon junctions of the following genes: ATM, BARD1, BRCA1, BRCA2, BRIP1, CDH1, CDK12, CHEK1, CHEK2, FANCA, FANCL, HDAC2, PALB2, PPP2R2A, PTEN, RAD51B, RAD51C, RAD51D, RAD54L, and TP53. Based on diagnostic criteria, clinical guidelines, relevant databases, and drug sensitivity evidence, the reported gene mutations were categorized into 4-tiered system: Tier I, variants of vital clinical significance; Tier II, variants of potential clinical significance; Tier III, variants deemd benign or likely benign. Germline mutations were further classified into five Tiers: TierI: Benign, TierII: Likely benign, Tier III: Uncertain significance, TierIV: Likely pathogenic, TierV: Pathogenic. Raw data from gene sequencing were subjected to splitting, quality control, and formatting before being aligned with the human reference genome. Variants, including Loss of Heterozygosity (LOH), Allelic Somatic Copy Number Variations (ASCNV), and Base Copy Number Variations (BCNV), were identified based on alignment results, chromosomal fragment lengths, variant types, and positions. Using a support vector machine (SVM) training model, the weight of each variant feature was calculated to derive the GSS score. Importantly, the interpretation threshold of GSS ≥ 50 was not arbitrarily set but is the predefined criterion of the AmoyDx HRD Detection Kit. This cutoff was established by the manufacturer based on an SVM algorithm integrating three key indicators of genomic instability—LOH, TAI, and LST, which has been validated in multiple studies (39, 46). HRD detection results were classified as follows: (1) Positive: GSS score ≥ 50; (2) Positive: GSS score < 50 but with I/II mutations in BRCA1 or BRCA2; (3) Negative: GSS score < 50 without I/II mutations in BRCA1 or BRCA2. HRD-positive was defined by BRCA1/2 mutation-positive or GSS ≥ 50. If the BRCA1/2 mutation was positive, germline verification was performed using the patient’s blood sample (26).

### 2.3 Data analysis

All statistical analyses were conducted using R software (Version 4.2.2). The chi-squared test, Fisher’s exact test, and Wilcoxon rank sum test were used to identify differences between HRD status and clinical characteristics. A two-sided *P* value of <0.05 was considered statistically significant for all analyses performed in this study.

### 2.4 Neoadjuvant therapy efficacy analysis

Among the overall cohort, patients who received neoadjuvant therapy were further evaluated for treatment efficacy. Only patients with complete pathological complete response (pCR) and Miller–Payne grading were included in the efficacy analysis; patients without complete efficacy endpoints were excluded.

## 3 Results

### 3.1 Overview of baseline characteristics of breast cancer patients with different HRD status

A total of 133 patients were included in the final analysis. Based on BRCA1/2 mutation status and GSS scores, the patients were categorized into four subgroups: (1) BRCA1/2+ & GSS ≥ 50; (2) BRCA1/2- & GSS ≥ 50; (3) BRCA1/2+ & GSS < 50; (4) BRCA1/2- & GSS < 50. According to the HRD definition, subgroups (1), (2), and (3) were classified as HRD-positive. Approximately 54.1% (72/133) of the patients were HRD-positive. Notably, 36.8% (49/133) of the patients had negative BRCA1/2 mutation tests but were still classified as HRD-positive (**Table 1**).

**Table 1:** Subgroups of breast cancer patients according to GSS and BRCA1/2 mutations.

BRCA1/2 mutation	GSS	HRD	N (%)
+	≥ 50	+	21 (15.8)
–	≥ 50	+	49 (36.8)
+	< 50	+	2 (1.5)
–	< 50	–	61 (45.9)

### 3.2 HRR pathway-related gene mutations in breast cancer with different HRD Status

**Figure 2:**
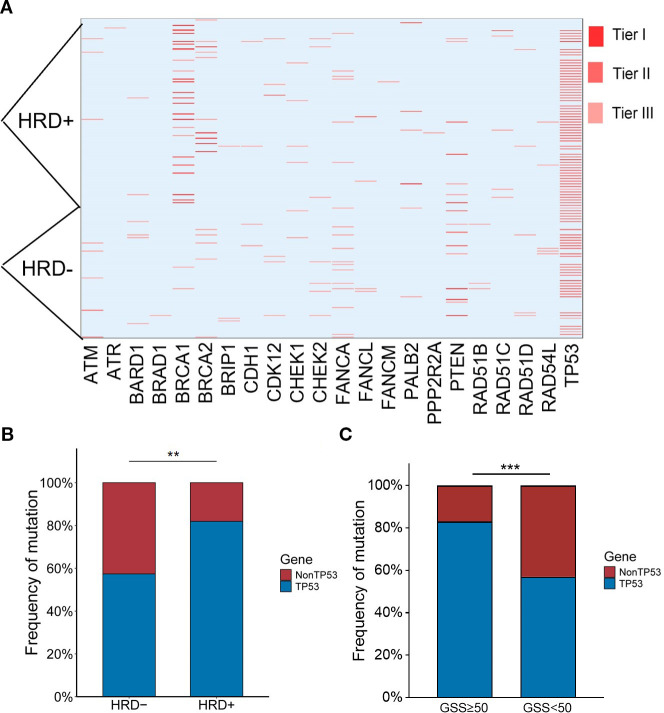
**(A)** Heatmap of HRR mutations. Tier I, variants of vital clinical significance; Tier II, variants of potential clinical significance; Tier III, variants deemed benign or likely benign. **(B)** Relationship between TP53 mutation rate and HRD status. **(C)** Relationship between TP53 mutation rate and GSS status. *p < 0.05, **p < 0.01, ***p < 0.001, ns, not significant.

Among the 133 enrolled patients, 93 (87.7%) harbored mutations in genes related to the HRR pathway. HRD was identified in 72 patients (54.1%), of whom 69 (95.8%) carried HRR-related gene mutations. In comparison, among the 61 HRD-negative patients, 50 (82.0%) also had HRR-related mutations. Within the HRD-positive group, 23 patients (31.9%) had BRCA1/2 mutations. Among the BRCA1/2-mutated cases, 14 (60.9%) were of germline origin (**Table 2**), whereas 9 (39.1%) were somatic (**Table 3**). The mutation landscape of HRR pathway genes in HRD-positive and HRD-negative patients is depicted in **Figure 2A**. TP53 mutations were the most prevalent in both groups. In HRD-positive patients, TP53 mutations were present in 81.94%, predominantly as missense variants, with occasional multi-hit events observed in individual genes. In contrast, TP53 mutations occurred in 57.38% of HRD-negative patients. This difference was statistically significant (*P* = 0.004) (**Figure 2B**). An additional analysis revealed that TP53-mutant tumors were significantly more likely to exhibit high GSS values (≥50) compared with TP53 wild-type tumors (*P* = 0.0004)(**Figure 2C**).

**Table 2:** Germline mutation in 23 BRCA1/2 mutation-positive patients.

NO.	Germline mutation	BRCA gene	Mutation site-protein	Mutation site-nucleic acid	Mutation position	Mutation type	Mutation classification
7	Germline	BRCA1	p.E489*	c.1465G>T	exon11	nonsense	5
8	Germline	BRCA1	p.Q910Kfs*90	c.2728del	exon11	frameshift-del	5
11	Germline	BRCA1	p.C64R	c.190T>C	exon5	missense	4
12	Germline	BRCA1	p.H513*	c.1535-1536insATGA	exon11	nonsense	4
15	Germline	BRCA1	p.N997Ifs*3	c.2990del	exon11	frameshift-del	5
26	Germline	BRCA1	p.W1718*	c.5154G>A	exon19	nonsense	5
31	Germline	BRCA1	p.F1571Sfs*30	c.4712del	exon16	frameshift-del	5
33	Germline	BRCA1	p.R1443*	c.4327C>T	exon13	nonsense	5
35	Germline	BRCA1	p.L1098Sfs*4	c.3288_3289del	exon11	frameshift-del	5
40	Germline	BRCA1	/	c.212-1G>T	intron5	intron splice	5
50	Germline	BRCA2	p.D687*	c.2059 2063del	exon11	nonsense	5
52	Germline	BRCA2	p.N1742Kfs*35	c.5226del	exon11	frameshift-del	4
60	Germline	BRCA1	p.L1098Sfs*4	c.3288_3289del	exon11	frameshift-del	5
	Germline	BRCA1	p.(E23Rfs*18)	c.66dup	exon2	frameshift-del	5

**Table 3:** Somatic mutation in 23 BRCA1/2 mutation-positive patients.

NO.	Somatic mutation	BRCA gene	Mutation site-protein	Mutation site-nucleic acid	Mutation position	Mutation type	Mutation classification
4	Somatic	BRCA1	p.I1824Dfs*3	c.5470_5477del	exon24	frameshift-del	1
13	Somatic	BRCA2	p.Q1987*	c.5959C>T	exon11	nonsense	1
26	Somatic	BRCA1	p.E489*	c.1465G>T	exon11	nonsense	1
40	Somatic	BRCA1	p.S1841Vfs*2	c.5521del	exon24	frameshift-del	1
46	Somatic	BRCA2	p.D2819H	c.8455G>C	exon19	missense	1
53	Somatic	BRCA2	p.Q84Lfs*18	c.250_251insTTGC	exon3	frameshift-del	1
55	Somatic	BRCA1	p.Q858*	c.2572C>T	exon11	nonsense	1
61	Somatic	BRCA1	p.E1210Rfs*9	c.3627dup	exon11	frameshift-del	1
71	Somatic	BRCA1	p.(C61R)	c.181T>C	exon5	missense	1

### 3.3 Clinical and pathological characteristics of breast cancer with different HRD status

**Figure 3:**
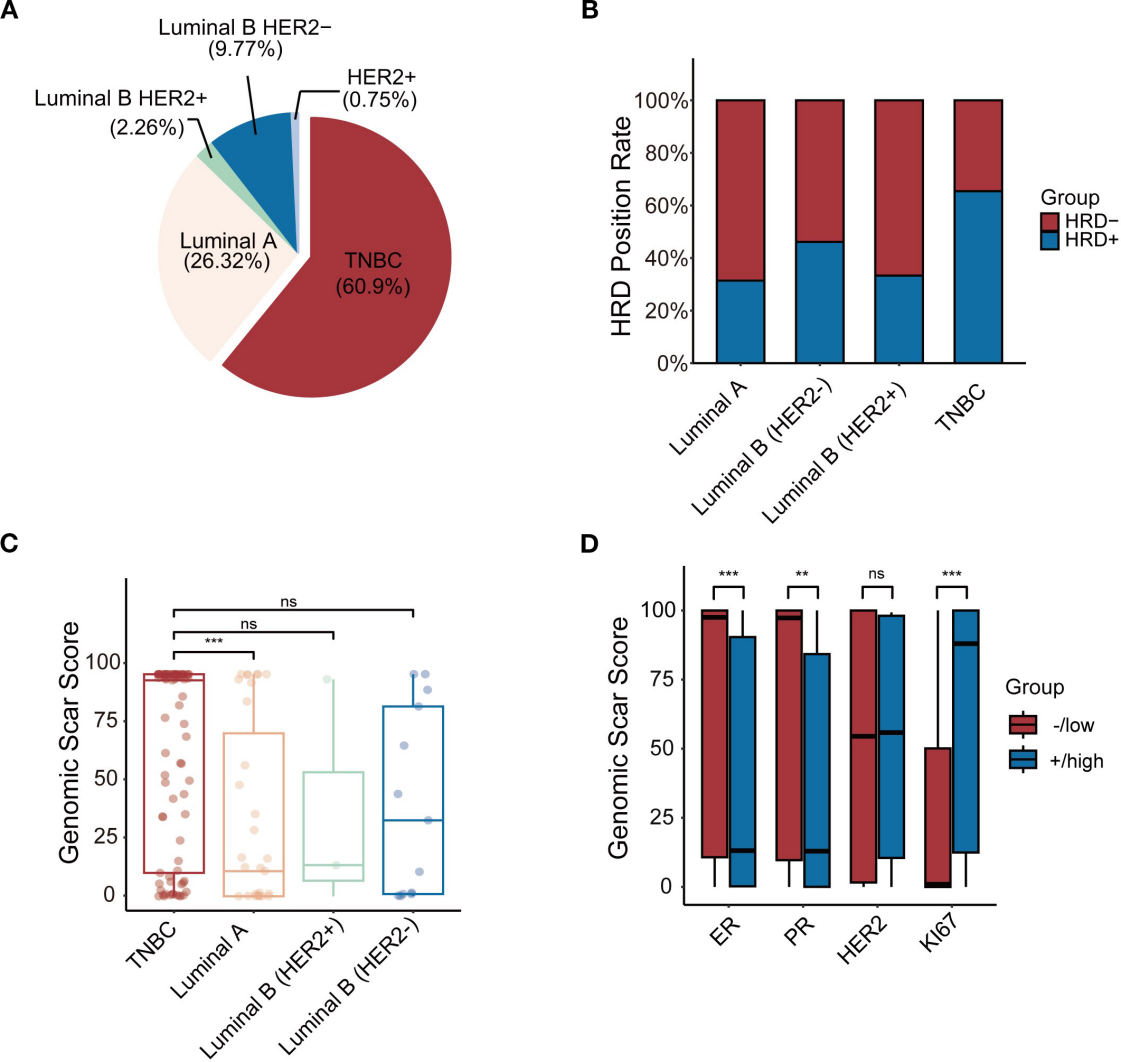
**(A)** The frequency of 5 subtypes of BC patients. **(B)** Comparison of positive rates of 4 molecular subtypes of HRD. **(C)** Comparison of GSS between molecular subtypes. **(D)** The relationship between GSS and IHC.*p < 0.05, **p < 0.01, ***p < 0.001, ns, not significant.

Between January 2021 and May 2024, 133 breast cancer patients underwent HRD testing (**Table 4**). The average age at diagnosis was 43 years overall, with HRD-negative patients having an average age of 43 years and HRD-positive patients slightly younger at 41 years. HRD-positive patients were more likely to exhibit low ER and PR expression levels (*P* < 0.001 and *P* = 0.004), higher Ki67 expression (*P* < 0.001), and higher T-stage (*P* = 0.025) compared to HRD-negative patients. Breast cancer was classified into five molecular subtypes based on ER, PR, HER2, and Ki67 expression (27): Luminal A: (26.32%, 35/133); Luminal B (HER2-) (9.77%, 13/133); Luminal B (HER2+) (2.26%, 3/133); TNBC(60.90%, 81/133); HER2+ (0.75%, 1/133) (**Figure 3A**). Due to the small number of HER2+ samples, this subtype was excluded from subgroup analyses.

**Table 4:** Clinical characteristics of breast cancer patients with HRD status.

Characteristics	Number of patients (%)	HRD- (%)	HRD+			*P* value
			Total (%)	BRCA1/2- (%)	BRCA1/2+ (%)	
	(n=133)	(n=61)	(n=72)	(n=49)	(n=23)	
Age of diagnosis						
Layer 1						0.143
< 35	30 (22.6)	11 (36.7)	19 (63.3)	14 (46.6)	5 (16.7)	
35-55	90 (67.7)	41 (45.6)	49 (54.4)	31 (34.4)	18 (20.0)	
> 55	13 (9.8)	9 (69.2)	4 (30.8)	4 (30.8)	0	
Layer 2						0.212
< 40	59 (44.4)	25 (42.4)	34 (57.6)	21 (35.6)	13 (22.0)	
40-60	66 (49.6)	30 (45.5)	36 (54.5)	26 (39.4)	10 (15.2)	
> 60	8 (6.0)	6 (75.0)	2 (25.0)	2 (25.0)	0	
Family history of cancer						0.495
No	101 (75.9)	48 (47.5)	53 (52.5)	37 (36.6)	16 (15.8)	
Yes	32 (24.1)	13 (40.6)	19 (59.4)	12 (37.5)	7 (21.9)	
Menopausal status						0.762
non-menopause	89 (66.9)	40 (44.9)	49 (55.1)	33 (37.1)	16 (18.0)	
menopause	44 (33.1)	21 (47.7)	23 (52.3)	16 (36.4)	7 (15.9)	
Side						0.994
Left	72 (54.1)	33 (45.8)	39 (54.2)	27 (37.5)	12 (16.7)	
Right	61 (45.9)	28 (45.9)	33 (54.1)	22 (36.1)	11 (18.0)	
Surgery type						0.995
Unoperated	15 (11.3)	7 (46.7)	8 (53.3)	6 (40.0)	4 (13.3)	
Improved radical surgery	51 (38.3)	24 (47.1)	27 (52.9)	20 (39.2)	7 (13.7)	
Others	65 (48.9)	30 (46.2)	35 (53.8)	23 (35.4)	12 (18.5)	
ER						<0.001
Negative	77 (57.9)	25 (32.5)	52 (67.5)	38 (49.4)	14 (18.2)	
Positive	55 (41.4)	35 (63.6)	20 (36.4)	11 (55.0)	9 (45.0)	
PR						0.004
Negative	86 (64.7)	30 (34.9)	56 (65.1)	41 (47.7)	15 (17.4)	
Positive	46 (35.3)	30 (65.2)	16 (34.8)	8 (50.0)	8 (50.0)	
HER2						0.713
0	71 (53.4)	29 (40.8)	42 (59.2)	32 (45.1)	10 (14.1)	
1+	42 (31.6)	21 (50.0)	21 (50.0)	15 (35.7)	6 (14.3)	
2+ FISH-	15 (11.3)	8 (53.3)	7 (46.7)	1 (6.7)	6 (40.0)	
2+ FISH+ or 3+	4 (3.0)	2 (50.0)	2 (50.0)	1 (25.0)	1 (25.0)	
Ki-67						<0.001
≤30%	37 (27.8)	26 (70.3)	11 (29.7)	6 (16.2)	5 (13.5)	
>30%	96 (72.2)	35 (36.5)	61 (63.5)	43 (44.8)	18 (18.8)	
AR						0.006
Not detected	38 (28.6)	19 (50.0)	19 (50.0)	15 (39.5)	4 (10.5)	
Negative	42 (31.6)	13 (31.0)	29 (69.0)	19 (45.2)	10 (23.8)	
Positive	53 (39.8)	29 (54.7)	24 (45.3)	15 (62.5)	9 (37.5)	
Molecular subtypes						0.001
Others	52 (39.1)	33 (63.5)	19 (36.5)	9 (17.3)	10 (19.2)	
TNBC	81 (60.9)	28 (34.6)	53 (65.4)	40 (49.4)	13 (16.0)	
T stage						0.025
T0	1 (0.8)	1 (100.0)	0	0	0	
T1 (≤ 2 cm)	41 (30.8)	25 (61.0)	16 (39.0)	9 (22.0)	7 (17.1)	
T2 (> 2 cm, < 5 cm)	67 (50.4)	24 (35.8)	43 (64.2)	31 (46.3)	12 (17.9)	
T3 (≥ 5 cm)	11 (8.3)	7 (63.6)	4 (36.4)	2 (18.2)	2 (18.2)	
T4	13 (9.8)	4 (30.8)	9 (69.2)	7 (53.8)	2 (15.4)	
N stage						0.842
N0	61 (45.9)	27 (44.3)	34 (55.7)	25 (41.0)	9 (14.7)	
N1	37 (27.8)	19 (51.4)	18 (48.6)	10 (27.0)	8 (21.6)	
N2	14 (10.5)	6 (42.9)	8 (57.1)	4 (28.6)	4 (28.6)	
N3	20 (15.0)	8 (40.0)	12 (60.0)	10 (50.0)	2 (10.0)	
Clinical stages						0.656
I	23 (17.3)	13 (56.5)	10 (43.5)	5 (21.7)	5 (21.7)	
II	57 (42.9)	24 (42.1)	33 (57.9)	22 (38.6)	11 (18.3)	
III	24 (18.0)	10 (41.7)	14 (58.3)	10 (41.7)	4 (16.7)	
IV	29 (21.8)	14 (48.3)	15 (51.7)	12 (41.4)	3 (10.3)	

In the TNBC subgroup, 73.6% (53/81) were HRD-positive compared to 26.4% in the HR+ group. This difference was statistically significant (*P* = 0.001). However, no significant differences were observed in HRD positivity among Luminal A, Luminal B (HER2-), Luminal B (HER2+), and HER2+ subtypes (**Figure 3B**). Subgroup analysis revealed that TNBC patients had significantly higher GSS scores compared to Luminal A patients (*P* = 0.001). However, no significant differences were observed when comparing TNBC with Luminal B (HER2+) (*P* = 0.341) or Luminal B (HER2-) patients (*P* = 0.219) (**Figure 3C**). Tumours with higher GSS scores were more likely to have low ER expression levels (*P* = 0.001) and PR expression levels (*P* = 0.002) and high Ki67 expression (*P* = 0.001) (**Figure 3D**).

**Figure 4:**
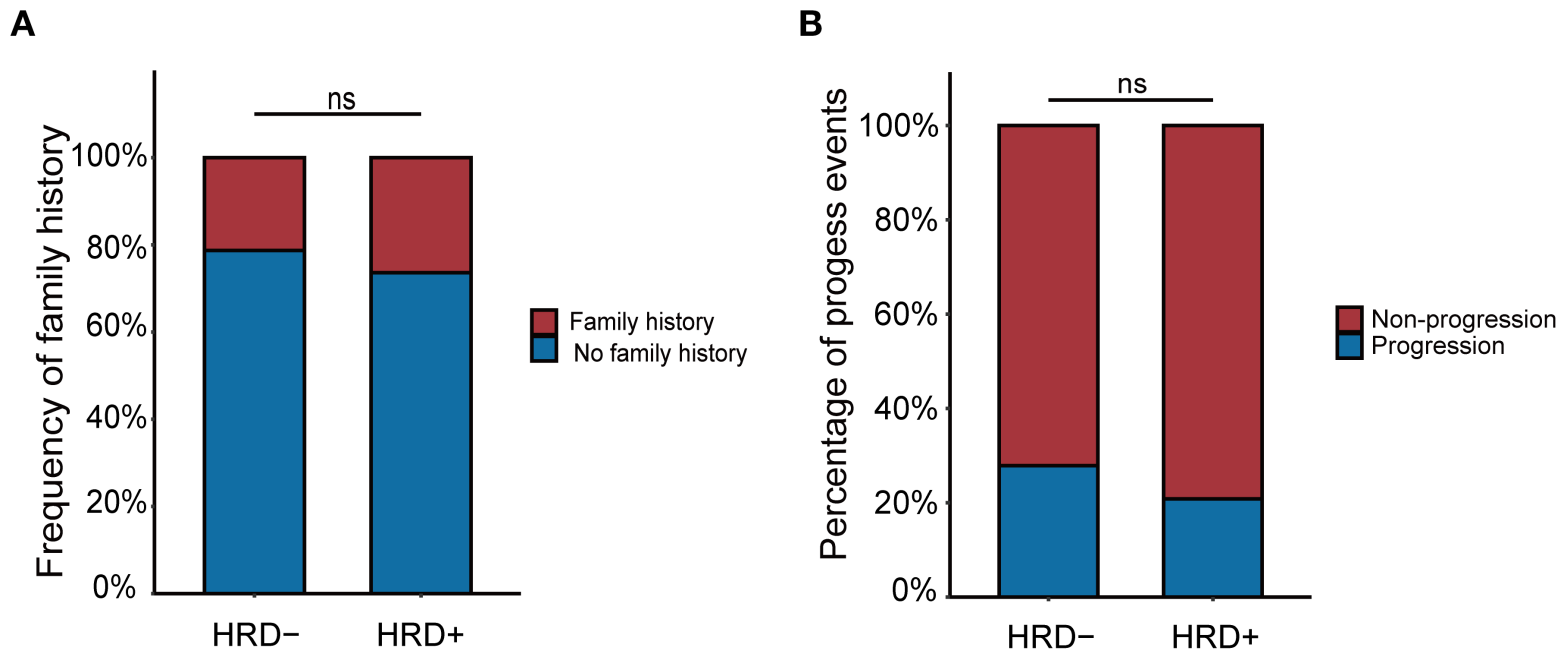
**(A)** The relationship between family history and HRD status. **(B)** The relationship between progression and HRD status.

A telephone follow-up was conducted to assess family cancer history. Among all patients, 47.7% (32/133) reported a family history of cancer. Of these, 59.4% (19/32) were HRD-positive, compared to 52.5% (53/101) of patients without a family history. However, this difference was not statistically significant (*P* = 0.495) (**Figure 4A**).

### 3.4 Efficacy analysis of neoadjuvant therapy in the study population

Among the 133 patients who underwent HRD testing, 54 received neoadjuvant therapy, including 44 TNBC cases. Within this NAT subgroup, clinical stages were distributed as stage I 5.6% (3/54), stage II 63.0% (34/54), and stage III 31.5% (17/54). Regardless of HRD status, no statistically significant differences were observed in the efficacy of neoadjuvant therapy based on either pathological complete response (pCR) or Miller-Payne (MP) grading (*P* = 0.158 and *P* = 0.284) (**Table 5**). The study indicated that HRD-positive breast cancer predominantly includes TNBC and ER+ subtypes. Neoadjuvant chemotherapy was more commonly administered in TNBC patients, whereas only high-risk ER+ patients required chemotherapy. To evaluate the hypothesis that HRD-positive tumors may be more sensitive to platinum-based DNA cross-linking agents, the efficacy of platinum-containing neoadjuvant chemotherapy was analyzed in HRD-positive and HRD-negative TNBC patients (**Table 6**). The results showed no statistically significant differences in the efficacy of platinum-based chemotherapy, regardless of HRD status (pCR: *P* = 0.648; MP grading: *P* = 0.361). Further analysis comparing platinum-based and non-platinum-based regimens in HRD-positive TNBC patients also revealed no significant differences (pCR: *P* = 0.715; MP grading: *P* = 0.713). However, HRD-positive TNBC patients treated with anthracyclines showed significantly higher pCR rates (*P* = 0.042), suggesting greater sensitivity to anthracycline-based therapies (**Table 7**). To further clarify whether this observed benefit could be attributed to the concomitant use of platinum, we performed a Fisher’s exact test comparing anthracycline-based regimens with and without platinum in HRD-positive patients. The difference in pCR rates between the two groups was not statistically significant (*P* = 0.467), indicating that the therapeutic advantage was unlikely to be solely driven by platinum co-administration(**Table 8**).

**Table 5:** Efficacy evaluation of neoadjuvant therapy for different HRD status in the whole population.

Efficacy evaluation	HRD+ (%)	HRD- (%)	*P* value
	(n=31)	(n=23)	
Whether pCR			0.158
No	16 (51.6)	17 (73.9)	
Yes	15 (48.4)	6 (26.1)	
Miller-Payne			0.284
I-III	14 (45.2)	14 (60.9)	
IV-V	17 (54.8)	9 (39.1)	

**Table 6:** Comparison of the efficacy of platinum-containing neoadjuvant therapy for TNBC with different HRD status.

Efficacy evaluation	HRD+ (%)	HRD- (%)	*P* value
	(n=15)	(n=7)	
Whether pCR			0.648
No	8 (53.3)	5 (71.4)	
Yes	7 (46.7)	2 (28.6)	
Miller-Payne			0.361
I-III	6 (40.0)	5 (71.4)	
IV-V	9 (60.0)	2 (28.6)	

**Table 7:** Comparison of the efficacy of neoadjuvant chemotherapy in HRD positive TNBC patients.

Efficacy evaluation	Platinum-free (%)	Platinum-based (%)	*P* value	Anthracycline-free	Anthracycline based	*P* value
	(n=15)	(n=15)		(n=5)	(n= 25)	
Whether pCR			0.715			0.042
No	7 (46.7)	8 (53.3)		5 (100.0)	10 (40.0)	
Yes	8 (53.3)	7 (46.7)		0	15 (60.0)	
Miller-Payne			0.713			0.138
I-III	7 (46.7)	6 (40.0)		4 (80.0)	9 (36.0)	
IV-V	8 (53.3)	9 (60.0)		1 (20.0)	16 (64.0)	

**Table 8:** Comparison of the efficacy of anthracycline monotherapy and anthracycline combined with platinum in HRD positive TNBC patients.

Efficacy evaluation	Anthracycline (%)	Anthracycline and Platinum (%)	*P* value
	(n=15)	(n=10)	
Whether pCR			0.467
No	7 (46.7)	3 (30.0)	
Yes	8 (53.3)	7 (70.0)	

### 3.5 Progression events and HRD status

At the cutoff date (May 31, 2024), we evaluated whether patients had experienced progression events during the observation window. A total of 32 patients experienced disease progression, including 15 (20.83%) HRD-positive and 17 (27.87%) HRD-negative patients (**Figure 4B**). Although HRD-negative patients were more likely to experience progression, the difference was not statistically significant (*P* = 0.458). Then we conducted a correlation analysis between HRD status and the events of disease progression among these patients (**Table 9**). The results revealed that, compared with HRD-negative patients, HRD-positive patients exhibited higher proportions of bone metastasis (46.7% vs. 23.5%), brain metastasis (13.3% vs. 6.3%), breast recurrence or metastasis (20.0% vs. 13.3%), and lymph node metastasis (46.7% vs. 29.4%). However, these differences did not reach statistical significance. Conversely, HRD-negative patients displayed a greater proportion of lung metastasis (52.9% vs. 26.7%), yet the statistical difference remained non-significant.

**Table 9:** Comparison of differences between progression sites and HRD status.

	HRD- (%)	HRD+ (%)	*P* value
	(n=17)	(n=15)	
Bone metastasis			0.169
Yes	4 (23.5)	7 (46.7)	
No	13 (76.5)	8 (53.3)	
Brain metastasis			0.909
Yes	1 (6.3)	2 (13.3)	
No	16 (93.7)	13 (86.7)	
Breast recurrence or metastasis			0.879
Yes	2 (13.3)	3 (20.0)	
No	15 (86.7)	12 (80.0)	
Liver metastasis			1
Yes	4 (23.5)	3 (20.0)	
No	13 (76.5)	12 (80.0)	
Lung metastasis			0.131
Yes	9 (52.9)	4 (26.7)	
No	8 (47.1)	11 (73.3)	
Lymph node metastasis			0.314
Yes	5 (29.4)	7 (46.7)	
No	12 (70.6)	8 (53.3)	

## 4 Discussion

Several *in vitro* diagnostic reagent companies, both at home and abroad, have developed HRD detection kits on the NGS platform. The calculation methods and cut-off values for these kits vary among different companies (28). The definitions and weights of various HRD indicators are inconsistent, algorithms lack standardization, and there is no scoring system tailored for Chinese patients (29). These are urgent issues that need to be addressed in HRD detection in China. Recently, during the IIa and IIb phases of the Chinese HRD Coordination Project, HRD reference materials and reference datasets covering multiple cancer types, such as lung cancer, breast cancer, and melanoma, have been successfully developed, providing reproducible and comparable standards for HRD analysis in NGS (next-generation sequencing) detection (30). Although there are currently no commercially available HRD detection products approved by the National Medical Products Administration (NMPA) in China, qualified and validated HRD kits are already accessible domestically (31). The detection method used in this study is the AmoyDx^®^ HRD Panel based on GSS. Up to now, central laboratories in multiple countries around the world have independently evaluated the consistency between this product and Myriad^®^ myChoice CDx, and highly consistent results have been obtained. The latest data show that the overall consistency between AmoyDx^®^ HRD Focus Panel and Myriad^®^ MyChoice CDx reaches 88.6% (32). Since the cost of a single HRD test is approximately 5,000 to 8,000 yuan, and only tertiary hospitals and third-party institutions can conduct this test, it limits patients’ choices for detection (33). Fortunately, China has piloted the inclusion of HRD testing in the Class B medical insurance catalogue. With the breakthrough of ctDNA liquid biopsy technology, the cost of HRD testing is expected to decrease by 40% within three years (34). This provides an opportunity for us to expand HRD test samples and conduct multi-center clinical validation in the future.

In our study population, several sporadic multi-hit events in single genes were observed among HRD-positive breast cancer patients. However, the small sample size (only three cases) and the dispersed mutation patterns (involving genes such as FANCA, BARD1, and TP53) limited our ability to conduct a detailed analysis. Recurrent alterations were mainly detected in BRCA1, TP53, and PTEN, with TP53 missense mutations predominating. The high TP53 mutation rate in HRD-positive patients in this study suggests that TP53 status may serve as a complementary biomarker for HRD detection, especially in the non-BRCA mutation population. Our additional analysis showed that TP53-mutant tumors were significantly more likely to exhibit high genomic scar scores (≥50) compared with TP53 wild-type tumors, supporting a strong association between TP53 mutations and genomic instability. This provides a rationale for including TP53 in the AmoyDx HRD panel, which was developed with consideration of the molecular features of Chinese breast cancers. Related studies have shown that TP53 missense mutations can affect the tetrameric conformation of p53, impair its ability to bind to transcriptional targets, fail to trigger p21, down-regulate apoptosis-related genes, and up-regulate proteins involved in the cell cycle process and DDR (35). Patients carrying germline pathogenic variants may have tumors enriched with TP53 defects, especially in DDR genes. It is believed that TP53 dysfunction is a core mechanism in BRCA1/2-related tumorigenesis (36). Consistent with this, our findings suggest that TP53 may indirectly contribute to HRD-related phenotypes by influencing BRCA1/2 function, although this requires mechanistic validation. At the same time, according to research by Song et al. at the Cancer Hospital of Fudan University, in the TNBC cohort, TP53 mutations are the most common, with a mutation frequency of 49.9% (37). The TP53 p.R175H mutation is a known hotspot mutation in the Chinese population, with a mutation frequency of over 2%. This also suggests the need for regional-specific analysis in HRD detection from the perspective of clinical research (38).

Currently, there are limited reports on the clinical and pathological characteristics of breast cancer in the Chinese population based on HRD status. Our study showed that the positive detection rate of HRD is 54.1%. This detection rate is higher than that reported by Feng et al. (34.7%),and the correlation between HRD status and clinicopathological characteristics is highly consistent (39). The population we screened for HRD testing mainly focuses on patients with TNBC and HR+ breast cancer, especially those with advanced stages and a high risk of recurrence and metastasis. This may have a direct relationship with the relatively high positive detection rate of HRD in our screening.However, our study did not show a statistically significant difference in the efficacy of neoadjuvant therapy and platinum-containing neoadjuvant therapy between the HRD-positive and HRD-negative groups. This may be due to the more conservative interpretation of pCR and MP grading at our institution.

TNBC has a poor prognosis, with high recurrence and mortality rates. Due to the high mutation frequency of BRCA1/2 genes in TNBC, BRCA gene mutations usually lead to HRD, making HRD a potential therapeutic target for triple-negative breast cancer. Therefore, HRD detection often focuses on TNBC (40). Currently, in China, there is insufficient evidence to support the use of HRD status as a predictor of platinum sensitivity in neoadjuvant therapy for early TNBC, and this issue remains controversial (41). For example, in the PrECOG 0105 single-arm study, patients who received six cycles of platinum-based neoadjuvant chemotherapy and had a higher HRD-LOH score (≥ 10) had a significantly higher RCB0/1 rate than those with a lower score (< 10) (*P* = 0.0026) (42). Similarly, the exploratory analysis of the GeparSixto study showed that patients with a higher HRD score (≥ 42) had a significantly higher pathological complete response (pCR) rate than those with a lower score (< 42) (*P* = 0.001) (43). However, the TBCRC030 study showed no significant difference in the pCR rate between patients with high and low HRD scores who received 12 weeks of neoadjuvant therapy based on cisplatin or paclitaxel (44). Our study explored the relationship between HRD status and the efficacy of neoadjuvant therapy in the TNBC population, as well as the response of HRD-positive patients to platinum-based regimens. Consistent with the TBCRC030 study, we found no significant difference in the pCR rate between HRD-positive and HRD-negative TNBC patients who received platinum-based neoadjuvant chemotherapy, but the data trend suggests that HRD-positive TNBC may be sensitive to platinum. This divergence may reflect heterogeneity in HRD assessment methods, as our study employed the AmoyDx HRD panel with a Genomic Scar Score cutoff of ≥50, whereas other trials applied different scoring systems. In addition, the relatively small number of TNBC patients receiving platinum in our cohort highlights the need for further validation in larger, more comprehensive studies.

For a long time, combined or sequential neoadjuvant chemotherapy based on anthracyclines and taxanes has been the standard treatment for early high-risk TNBC (45). Interestingly, our study found that HRD-positive patients achieved higher pCR rates with anthracycline-containing neoadjuvant therapies, consistent with the findings of Professor Cao Wenming’s team in 2024. Cao’s study introduced a new HRD scoring algorithm, AcornHRD, designed for the genomic characteristics of the Chinese population, which showed a significant correlation between HRD scores and anthracycline sensitivity (46). These results support the recommendation of anthracycline-based regimens as the standard neoadjuvant treatment for TNBC in Chinese Clinical Oncology Association (CACA-CBCS) guidelines (47). Our analysis further indicated that the improved response observed in HRD-positive TNBC patients was unlikely to be solely attributable to platinum co-administration, suggesting a potential anthracycline-specific effect. Given the limited representation of patients not receiving anthracyclines, these findings should be regarded as exploratory. Future studies with larger, multi-center cohorts and adequately powered control groups will be essential to validate the independent contribution of anthracyclines and to refine HRD-guided treatment strategies.

## 5 Conclusion

This study applied an HRD detection product, developed based on the genomic characteristics of the Chinese population, to assess HRD status in breast cancer patients at a single center in China. Descriptive statistics were used to analyze clinicopathological features, family history, neoadjuvant efficacy, and disease progression across different HRD statuses, with particular emphasis on the impact of HRD status on the efficacy of platinum-based and anthracycline therapies in TNBC patients. The results confirm the effectiveness and feasibility of this HRD detection method, providing clinical evidence for its application. It is hoped that HRD screening will be expanded, particularly among TNBC patients, to support comprehensive tumor evaluation and optimize treatment strategies in future.

## Data Availability

The original contributions presented in the study are included in the article/supplementary material. Further inquiries can be directed to the corresponding authors.
